# Increase in brain connectivity with methylphenidate treatment in boys diagnosed with attention deficit hyperactivity disorder: A coherence-based qeeg analysis

**DOI:** 10.1192/j.eurpsy.2021.1686

**Published:** 2021-08-13

**Authors:** F.H. Çetin, Ş. Gıca, M. Çıkılı Uytun, Z. Babadağı, M.B. Usta, A.S. Güven, Y. Işık

**Affiliations:** 1 Child And Adolescent Psychiatry, Selcuk University, Faculty of Medicine, Konya, Turkey; 2 Psychiatry, Necmettin Erbakan Univesity, Meram Faculty of Medicine, Konya, Turkey; 3 Child And Adolescent Psychiatry, Ankara University, Faculty of Medicine, Ankara, Turkey; 4 Child And Adolescent Psychiatry, Kayseri City Hospital, Kayseri, Turkey; 5 Child And Adolescent Psychiatry, Ondokuz Mayıs University, Faculty of Medicine, Samsun, Turkey; 6 Pediatric Neurology, Necmettin Erbakan Univesity, Meram Faculty of Medicine, Konya, Turkey; 7 Child And Adolescent Psychiatry, Gazi University, Faculty of Medicine, Ankara, Turkey

**Keywords:** ADHD, methylphenidate, qEEG, connectivity

## Abstract

**Introduction:**

Attention deficit hyperactivity (ADHD) disorder is a common childhood neurodevelopmental disorder, and Methylphenidate (MPH) is a first-line therapeutic option for treating ADHD.However, how brain connectivity changes with methylphenidate treatment have yet to be studied.

**Objectives:**

This study investigates how the MPH treatment affects the connectivity in the brain of children with ADHD by coherence-based qEEG analysis during rest.

**Methods:**

During eyes-open resting, EEG signals were recorded from 25 boys with ADHD-combined type before MPH administration and at the end of the 1st month of the treatment. Mutual Information (MI),Coherence Function (COH) and Phase Locking Value (PLV) were used to analyse the changes in brain connectivity.

**Results:**

A statistically significant increase in connectivity level was found with MPH treatment between the F3-F4 channels, P3-P4 channels, F7-F8 channels and T5-T6 channels according to PLV, COH and MI analysis (p<0.001).

**Figure:**
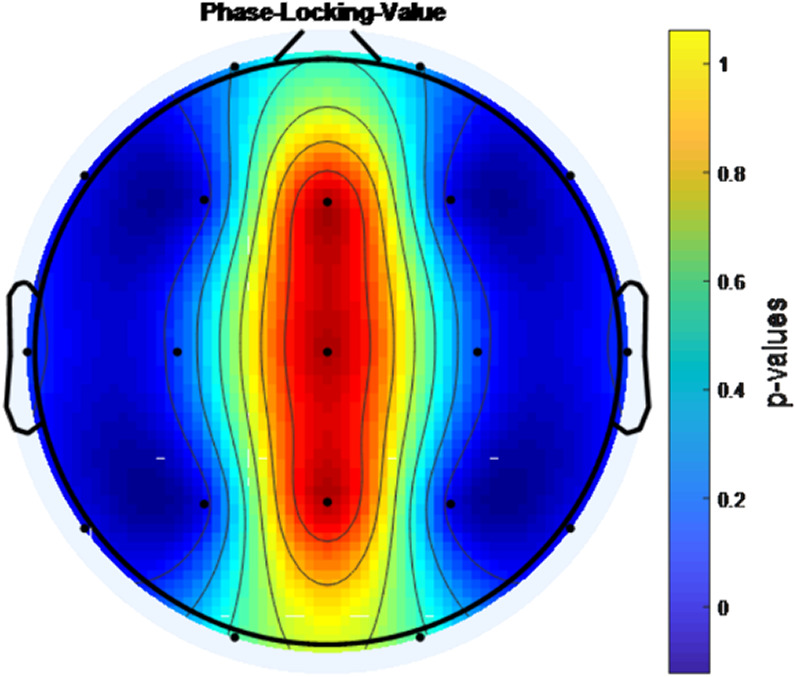

**Figure:**
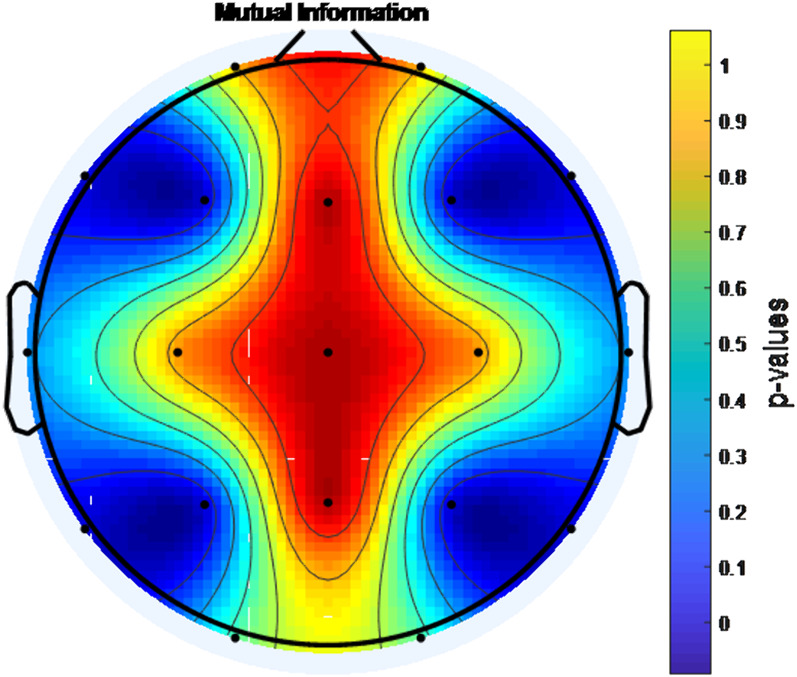

**Figure:**
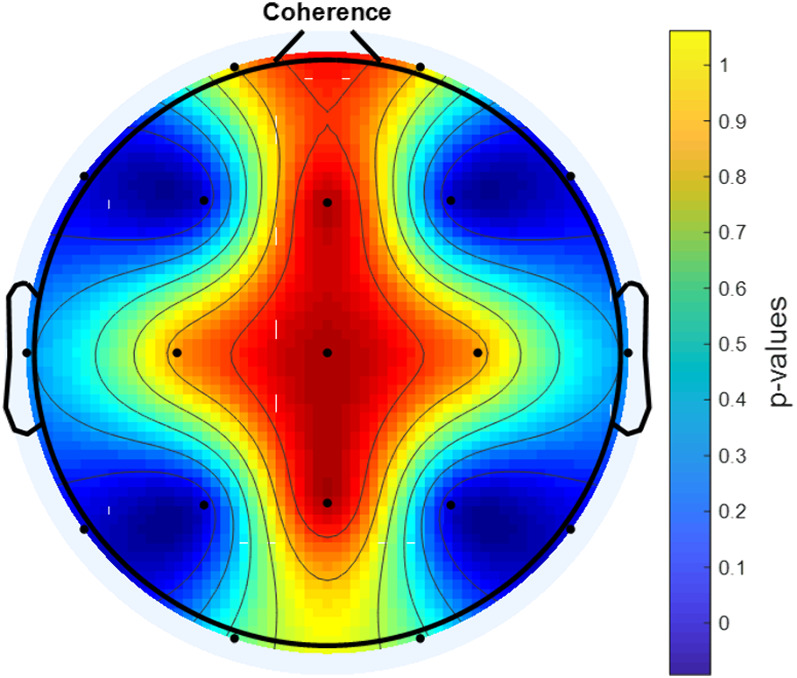

**Conclusions:**

This is the first study to investigate how MPH treatment affects the connectivity of the brain of children with ADHD. Coherence-based qEEG analysis may be a new method that can be used in diagnostic, clinical and prognostic predictions in ADHD.

**Disclosure:**

No significant relationships.

